# A curated census of autophagy-modulating proteins and small molecules: Candidate targets for cancer therapy

**DOI:** 10.4161/auto.28773

**Published:** 2014-05-12

**Authors:** Philip L Lorenzi, Sofie Claerhout, Gordon B Mills, John N Weinstein

**Affiliations:** 1 Department of Bioinformatics and Computational Biology; The University of Texas MD Anderson Cancer Center; Houston, TX USA; 2 Department of Systems Biology; The University of Texas MD Anderson Cancer Center; Houston, TX USA

**Keywords:** autophagy, cancer, high-throughput screening, L-asparaginase, natural language processing, pathway analysis, RNAi, siRNA, text-mining

## Abstract

Autophagy, a programmed process in which cell contents are delivered to lysosomes for degradation, appears to have both tumor-suppressive and tumor-promoting functions; both stimulation and inhibition of autophagy have been reported to induce cancer cell death, and particular genes and proteins have been associated both positively and negatively with autophagy. To provide a basis for incisive analysis of those complexities and ambiguities and to guide development of new autophagy-targeted treatments for cancer, we have compiled a comprehensive, curated inventory of autophagy modulators by integrating information from published siRNA screens, multiple pathway analysis algorithms, and extensive, manually curated text-mining of the literature. The resulting inventory includes 739 proteins and 385 chemicals (including drugs, small molecules, and metabolites). Because autophagy is still at an early stage of investigation, we provide extensive analysis of our sources of information and their complex relationships with each other. We conclude with a discussion of novel strategies that could potentially be used to target autophagy for cancer therapy.

## Introduction

The term “autophagy” was coined in the 1960s to describe a “self-eating” process in which cell constituents are delivered to lysosomes for degradation.^1^ Autophagy has been divided into 3 major classes: macroautophagy, microautophagy,^2^ and chaperone-mediated autophagy.^3^ Because macroautophagy (which includes organelle-specific subpathways such as mitophagy) is by far the most common, we focus on it here, referring to it generically as “autophagy.” Autophagy plays a context-dependent role in cancer, as explained in a recent elegant review.^4^ Elimination of damaged cellular components through autophagy suppresses tissue injury and tumor initiation. However, in an established tumor, autophagy promotes cancer progression by providing substrates for metabolism, maintaining functional mitochondria, and fostering survival during and after therapy.

Although autophagy has been subjected to intensive investigation, the complex networks that regulate the process in human diseases have only begun to be elucidated. One recent report described the combined use of protein expression, immunoprecipitation, and mass spectrometry to identify an “Autophagy-Interaction Network” composed of 409 candidate interacting proteins with 751 discrete interactions.^5^ Another report described the use of mass spectrometry to identify 728 proteins apparently associated with autophagosomes.^6^ There is also an autophagy database.^7^ However, for identification of candidate cancer drug targets, it is important to assess the functional contribution of each protein to the modulation of autophagy, not just its apparent association with the process. In this analysis, we focused first on the identification of causal relationships through reanalysis of 4 macroautophagy-specific human cell line siRNA screens.^8^^-^^11^ However, there were limitations and complexities to interpretation of the siRNA screening data (see the Data Quality Assessment sub-section). The Venn diagram in Figure 1A shows the sizes of the siRNA libraries and the numbers of hits in each screen. Clearly, the correspondence of results was only moderate, in part because autophagy was defined by different end-points, in different cell types, and using different methods.

**Figure:**
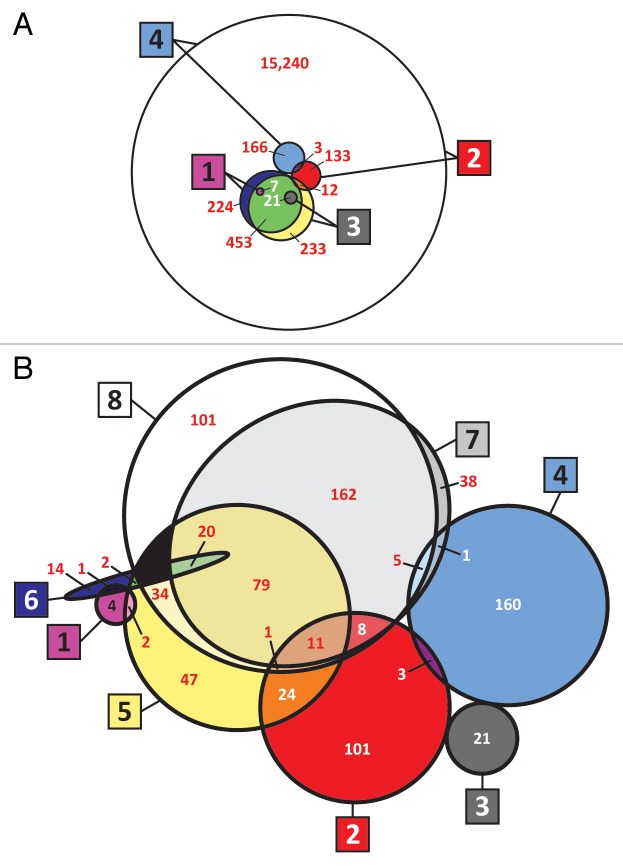
**Figure 1.** Venn diagrams (drawn approximately to scale) of the data sets used in the census. (**A**) siRNA libraries and hits, and (**B**) combined siRNA and pathway analysis results (hits). Large boxed numbers identify the set, and smaller white or red numbers indicate the number of genes or hits in the intersection set. 1: siRNA screen 1; 2: siRNA screen 2; 3: siRNA screen 3; 4: siRNA screen 4; 5: Ingenuity Pathway Analysis (IPA); 6: MetaCore; 7: Pathway Studio (raw hits); and 8: Pathway Studio (manually curated hits). Diagrams with all circles were generated using VennMaster 0.37.5 for calculations^12^^,^^13^ and then overlaying smooth circles on the VennMaster graphics. When 4 or more sets are being shown as circles, it is not in general mathematically possible to represent them and their intersections graphically to scale with accuracy. VennMaster provides an optimization algorithm that achieves a compromise representation. However, the 8 sets overlap in such a complex way that the fit could be improved by manually changing the circles for sets 6 and 7 to ellipses of approximately the right dimensions. In (**A**), the larger circle in each case represents the library, and the smaller circle represents validated hits. In (**B**), some small regions that contain zero hits (colored black) were necessary for graphical purposes.

We next complemented analysis of the siRNA screens with extensive pathway analyses of the relevant literature, using MetaCore (GeneGo; Thomson Reuters), Ingenuity Pathway Analysis (IPA; Ingenuity Systems), Pathway Studio (Elsevier/Ariadne Genomics) and our own extensive curation. The results were even less concordant (Fig. 1B), perhaps indicating the unsettled, complex nature of autophagy and its many functional relationships with important cellular processes. Therefore, to provide the most comprehensive list of candidate autophagy modulators possible, we focused on the union of the various sets in Figure 1B. Recognizing the likelihood of false-positives, however, we have compiled the lists in a series of heavily annotated **Supplementary Tables** for investigators who wish to minimize false positives at the risk of missing some true positives or molecules that modulate autophagy in context-dependent ways. The individual data sets (or any Boolean combination thereof) can be interrogated to assess the nature of the evidence for a candidate molecule. To provide crude indices of the likelihood that a candidate is a true positive, we list the number of siRNA screens in which it was identified and its rank in terms of literature references that support it as a modulator of autophagy. Details of this complex analysis are presented in the section on methodology of the analysis, after we survey the molecules identified as candidate modulators. Our principal aim is to highlight candidate targets for autophagy-related therapy of cancer.

### Integrated analysis of candidate autophagy-modulating genes

The union of siRNA and text-mining data yielded 739 apparent autophagy-modulating entities (proteins and complexes). A truncated subset of the top-ranked hits is provided in Table 1. We parsed the full set of results into 7 tables (Table S1A–S1G), with each entity appearing in only 1 table according to its direction of autophagy modulation (positive or negative). A Venn diagram of the integrated siRNA-pathway analysis results (Fig. 2A) serves as a guide. We also present a detailed pathway schematic of the autophagy process that puts many entities from the census in their functional contexts (Fig. 3). Since one goal of this analysis was to highlight potential targets and strategies for treatment of cancer, we focus our discussion in each section below on the therapeutic potential of top-ranked autophagy modulators.

**Table:** **Table 1.** Top-ranked autophagy-modulating genes, proteins, and protein complexes

Gene symbol/entity name	Direction of modulation	Data set	Supplemental Table	# of REFs^a^	Rank^b^
MTOR	Negative	Text	S2A	137	1
AKT1				76	2
TORC1 complex				55	3
INS				29	4
CASP8				12	5
BECN1 (ATG6)	Positive		S2B	130	1
BNIP3				58	5
NRBP2	Negative	siRNA 1	S2D	-	1
CDK8				-	2
XPO1		siRNA 2		-	1
PTPRU				-	2
GHSR		siRNA 2 | siRNA 4		-	51 | 65
CHAF1B				-	56 | 78
CSNK1A1		siRNA 3		-	1
MAST2				-	2
GAB1		siRNA 4		-	1
KREMEN2				-	2
PLBD1	Positive		S2E	-	1
LRRN4				-	2
MAP2K6		siRNA 1		-	1
HUNK				-	2
NFKB1		siRNA 2		-	2
CASP1	Negative	text | siRNA 2	S2F	4	27 | 102
EP300				3	160 | 18
IGF1				7	15 | 58
STAT3				3	160 | 92
ATG5	Positive		S2G	128	2 | 3
ATG7				95	3 | 1
RELA				3	116 | 5
ULK1 (ATG1)		text | siRNA 1		93	4 | 4

Top-ranked text-mined and siRNA hits (autophagy modulators), including those identified in more than one siRNA or text screen. “Text” represents text-mined data (from Pathway Studio), and “siRNA” represents results from the indicated siRNA screen. ^a^Number of references (for text-mined entries). ^b^Text and siRNA screen ranks reflect rank within corresponding screen (siRNA or text); additional information is available in Table S1.

**Figure:**
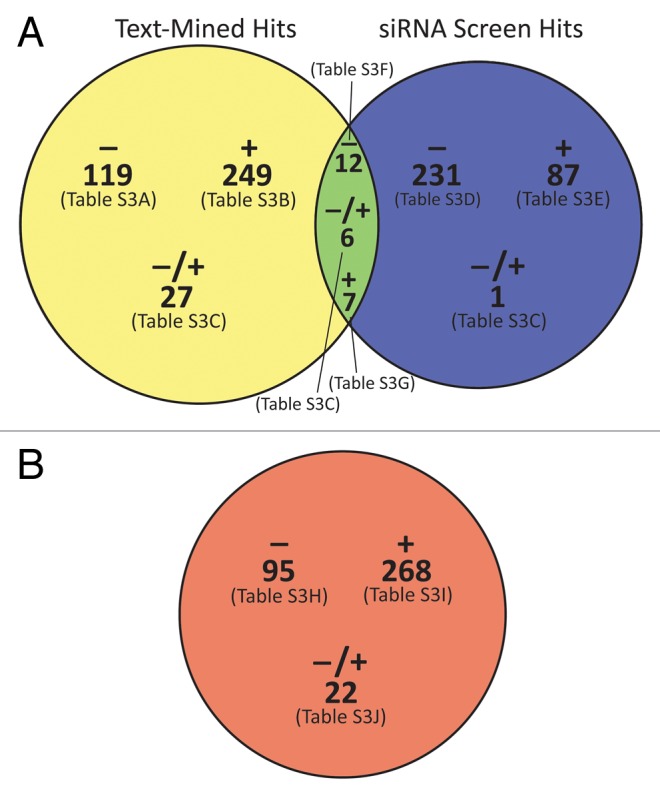
**Figure 2.** Venn diagrams of the integrated siRNA and pathway analysis results. (**A**) 739 total genes, proteins, and protein complexes were identified as apparent modulators of autophagy by text mining with our manual curation (yellow circle) and/or siRNA screening (blue circle). The diagram shows the classification of those entities into negative, positive, and dual-potential modulators, and it indicates the tables in Table S1 in which those entities are listed. (**B**) 385 small molecules were identified by text-mining as modulators of autophagy and categorized in the same manner. Circles were drawn approximately to scale using VennMaster as described in the legend to Figure 1.

**Figure:**
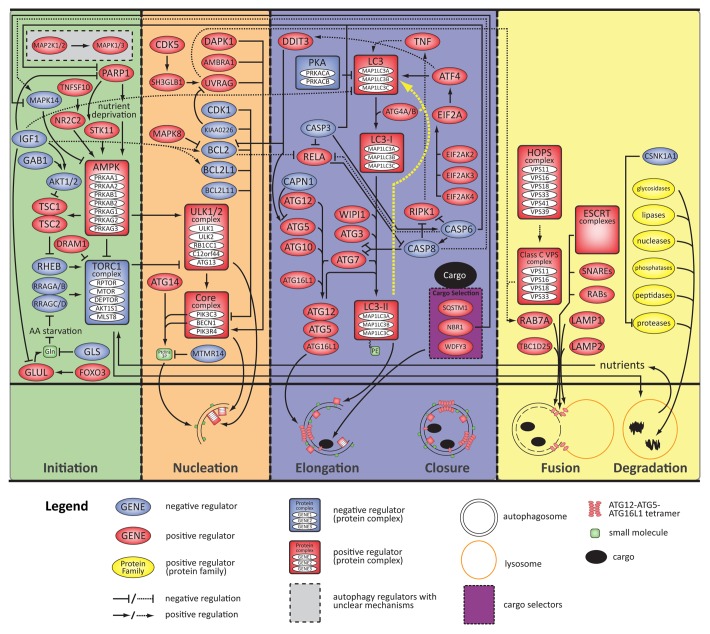
**Figure 3.** A molecular schematic of the autophagy process based on the information in this census. The top panel shows specific entities (genes, proteins, complexes, and small molecules) associated with the process and, to the extent possible, their specific roles. The middle panel shows a schematic timeline of the 4 stages of macroautophagy from initiation through degradation (colored sections separated by vertical dashed lines). The 3 small molecules depicted are glutamine (Gln), phosphatidylinositol 3-phosphate (PtdIns3P), and phosphatidylethanolamine (PE). Three established cargo selectors include SQSTM1/p62, NBR1, and WDFY3/ALFY. The yellow dotted line in the elongation/closure stage indicates recycling of PE and LC3 by ATG4 following degradation.

#### Positive modulators of autophagy

Text-mining and siRNA screening led, respectively, to the identification of 288 and 96 positive modulators (including dual positive-negative modulators) of autophagy. There were 7 entities in the intersection of the 2 sets (Fig. 2A; Table S1). Of those 7 consensus genes (listed in Table S1G), 2—*KIF5B* and *RELA*—have been reported to modulate cell death, mainly negatively,^14^^,^^15^ suggesting that inhibiting them may induce cell death. No viable inhibitors of KIF5B were found by text mining the scientific literature, but a number of RELA inhibitors have been reported. In particular, text mining helped us identify a report in which ~50% inhibition of RELA activity was observed after treatment with the antioxidant acetylcysteine (Table S1H).^16^ Another report identified sesquiterpenes as inhibitors of RELA.^17^ Hence, acetylcysteine or sesquiterpenes could be used to test the hypothesis that inhibiting autophagy through inhibition of RELA can induce cancer cell death. However, the effect of RELA on autophagy may vary depending on the nature of the DNA damage-inducing stimulus.^18^

#### Negative modulators of autophagy

Text-mining and siRNA screening led to the identification of 160 and 248 negative modulators (including dual positive-negative modulators) of autophagy, respectively, with 12 entities in the intersection of the 2 sets (Fig. 2A; Table S1). To further prioritize candidate drug targets within that overlapping set of 12 autophagy modulators (listed in Table S1F), we reasoned that the best candidates are those whose inhibition (by a drug) is expected to result in cell death. Eight of the 12 proteins—AURKA, CLCF1, CXCL12, EP300, FGFR1, IGF1, LIF, and SOD1—have been reported to modulate cell death negatively, suggesting that inhibition of those proteins might induce cell death. To assess those 8 proteins as possible drug targets, we used Pathway Studio to analyze their pathway relationships, identifying IGF1 as a key node with connections to 7 of the other entities (Fig. S1). The possibility that inhibition of a highly connected pathway node could have a greater effect than inhibition of a less connected one prompts the hypothesis that inhibition of IGF1 could have a greater effect than inhibition of 1 of the other targets. Furthermore, text-mining also identified 3 agents (acrylonitrile, tempol, and UO126) that have been reported to inhibit IGF1 and also 1 of the other 7 proteins. Of those, UO126, which is frequently used as a MAP2K1/MEK1 inhibitor but also inhibits IGF1^19^ and EP300,^20^ stands out as the top candidate to test the hypothesis that stimulating autophagy through inhibition of IGF1 and EP300 could induce cell death in established cancers. Additionally, stimulation of autophagy with UO126 might represent a viable cancer prevention strategy, as autophagy in normal cells would be expected to suppress the initiation of cancer through elimination of damaged proteins and abnormal mitochondria and by preventing accumulation of DNA damage.^4^

Several interesting negative modulators of autophagy were identified by siRNA screening alone (Table S1D). Notably, CSNK1A1 was the highest ranking hit from siRNA screen 3, and 2 additional casein kinase (CSNK) family members, CSNK1G2 and CSNK2A2, were found to be negative modulators of autophagy by siRNA screening (Table S1D), suggesting casein kinases as possible targets. Currently available CSNK inhibitors include: 1) heparin, which has been reported to inhibit both CSNK1A1^21^ and CSNK2A2,^22^ 2) ionomycin, which has been reported to inhibit CSNK1A1,^23^ and 3) suramin,^22^ 2-aminopurine,^24^ and 6-dimethyladenine,^24^ which have been reported to inhibit CSNK2A2. Hence, those agents alone or in combination with other drugs could be used to test the hypothesis that stimulating autophagy through inhibition of casein kinases results in cancer cell death.

#### Dual modulators of autophagy

An unexpected result of our analysis was the number of genes with data supporting both positive and negative modulation of autophagy (Table S1C). Those genes necessitated additional investigation to probe the apparent discrepancies. We found several genes in Table S1C—*AGER*, *GNAI1*, *TLR3*, and *TNF*—to be positive modulators by text mining yet negative modulators by siRNA screens. Several lines of evidence, however, support the conclusion that those genes are actually positive modulators of autophagy (i.e., that the text-mined results are more persuasive). First, Pathway Studio identified multiple references supporting positive modulatory roles for *AGER* and *TNF*. Furthermore, *TNF* is a positive modulator of the cell death pathway known as necroptosis, which is negatively modulated by *CASP8*.^25^^-^^29^ Since *CASP8* is expressed in only 16% of neuroblastoma cell lines,^30^ it is possible that the H4 neuroblastoma cell line used in siRNA screen 2 was deficient in *CASP8* or another negative modulator of necroptosis, rendering the H4 line susceptible to uncontrolled necroptosis.^28^ Silencing TNF in such a context would alleviate uncontrolled necroptosis and might induce autophagy to facilitate recovery. That explanation may extend to the discrepancies observed for *AGER*, *GNAI1*, and *TLR3* as well, since those discrepancies were also associated with siRNA screen 2 and the H4 cell line. Overall, each of the aforementioned genes may positively modulate autophagy under most circumstances, and the cell line used in siRNA screen 2 may simply represent an unusual genetic context in which those genes act as negative modulators of autophagy. These observations suggest the importance of conducting siRNA screens against multiple cell types in order to corroborate results across multiple genetic contexts and, ultimately, to reduce contradictory observations when merged with text mining results. The inherent sampling of data from a wide range of sources and cell types is an advantage of text mining.

For another set of dual autophagy modulators in Table S1C—*BAK1*, *BIRC5*, *CCL2*, *CTSD*, *CXCR4*, *DDIT3*, *E2F1*, *ERN1*, *GNAI3*, *HSPA5*, *HSPB8*, *IL6*, *LAMP2*, *NAMPT*, *NUPR1*, *PARP1*, *PINK1*, *PLD1*, *PPARG*, *RB1*, and *RICTOR*—at least one of the relationships was supported by only one text reference. In each case, analogous to the discussion of the H4 cell line above, the single reference may have been derived from context-dependent analytical conditions not reflected in other studies or from an unusual genetic context that may involve rewiring of autophagy and metabolic pathways.^31^ For example, *BAK1* is reported to be a negative modulator of autophagy only in the experimental context of dual *BAK1* and *BAX* knockdown;^32^ *BAK1* should therefore generally be considered a positive modulator of autophagy. On the other hand, another gene, *E2F1*, is identified as a negative autophagy modulator by just one reference,^33^ but the mechanism appears to be physiologically relevant. GNAI3 is a GTPase that negatively modulates autophagy in the active, GTP-bound form and positively modulates autophagy in the inactive GDP-bound form^34^—a pattern also shown by RRAGA/B and RRAGC/D.^35^ Like RRAGA/B and RRAGC/D, GNAI3 should be considered a negative modulator of autophagy, even though there is only a single reference to it as a negative modulator. *RRAGA/B* and *RRAGC/D*, incidentally, appear to be false negatives that were not identified by either text mining or siRNA screening. Overall, for the aforementioned set of 21 genes, the assignment of positive or negative modulation of autophagy is generally in favor of one direction, as discussed, but the possibility of anomalous modulation should be kept in mind. For the next set of genes, the conclusions are even less clear.

Nine entities identified as dual modulators of autophagy did not appear to exhibit a dominant direction of autophagy modulation in the analysis—*BAX*, *BCL2*, *BCL2L1*, *BCL2L11*, *FBXL20*, *MAPK14*, *MYC*, *PRKCD*, and *TP53* (Table S1C). Nevertheless, some clarification can be provided. The first gene, *BAX*, is reported to be a negative modulator of autophagy only in the experimental context of dual *BAK1* and *BAX* knockdown;^26^^,^^32^ hence, negative modulation of autophagy by *BAX* may occur only in that rare genetic context, which may not occur frequently in clinical tumors. *BAX* should therefore generally be considered a positive modulator of autophagy. For *BCL2*, *BCL2L1*, and *BCL2L11*, further investigation of the associated references suggested that positive modulation of autophagy is likely to be anomalous and, in some cases, erroneous. For example, one review article indicates that *BCL2L11* induces autophagy,^27^ but the original reference^25^ does not support that statement. The error was propagated through subsequent review articles, thereby generating supporting references. Thus, the 3 *BCL2* family members are likely to be negative modulators of autophagy. The next gene, *FBXL20*, was found to be a negative modulator of autophagy in siRNA screen 2,^9^ yet a positive modulator of autophagy in siRNA screen 4.^11^ Analogous to previous explanations of rare genetic contexts, this is another case that could be explained by the H4 neuroblastoma cell line containing a rare mutation. The result from screen 4 is therefore likely to be correct, or at least more generalizable. *MAPK14* exhibits stronger evidence favoring a negative modulatory role in autophagy; a recent publication describes a mechanism through which MAPK14 negatively modulates autophagy via phosphorylation of ATG5.^28^ The next gene, *MYC*, is probably a true dual modulator whose direction of autophagy modulation also depends on genetic context. *PRKCD* was subject to text-mining errors; for example, one reference from which positive modulation of autophagy was derived was actually a reference to *PRKCQ*.^29^ Hence, *PRKCD* is probably a negative modulator of autophagy. Finally, the dual modulation reported for *TP53* appears to be attributable to cellular localization; nuclear TP53 activates stress-induced autophagy genes transcriptionally,^30^ whereas cytoplasmic TP53 inhibits basal autophagy by an unknown mechanism.^36^ Overall, the ambiguous relationships of dual modulators of autophagy suggest that they are not likely to be good drug targets if the aim is to modulate autophagy.

### Small-molecule modulators of autophagy

Just as we used Pathway Studio to compile a list of autophagy-modulating genes, proteins, and complexes, we next used Pathway Studio to identify 385 small molecule modulators of autophagy: 95 negative modulators (Table S1H), 268 positive modulators (Table S1I), and 22 compounds that have been reported to modulate autophagy both negatively and positively (Table S1J). A Venn diagram summarizes the results (Fig. 2B), and a truncated subset of top-ranked positive and negative hits is provided in Table 2. Together with the genes, proteins, and complexes discussed previously, the identification of small molecule modulators of autophagy completes our census.

**Table:** **Table 2.** Top-ranked autophagy-modulating chemicals from literature searches

Entity name	Direction of modulation	Supplemental Table	# of REFs^b^	Rank^c^
Amino acids	Negative	S3H	157	2
Chloroquine			64	3
Bafilomycin A_1_			41	5
Nitrogen			39	6
Adenosine			17	8
AMP			11	12
Acetylcysteine			8	16
Hydroxychloroquine			8	16
Okadaic acid			8	16
Oxygen			8	16
Rapamycin	Positive	S3I	149	1
Reactive oxygen species (ROS)			97	2
Resveratrol			57	3
Ceramides			44	4
Calcium			38	5
Lithium			34	6
Oridonin			24	8
PtdIns3P^a^			24	8
Temozolomide			21	9
H_2_O_2_			19	12

Top-ranked text-mined hits (autophagy modulators) from Pathway Studio. ^a^Phosphatidylinositol 3-phosphate. ^b^Number of references (for text-mined entries). ^c^Ranks are based on the number of references; additional information is available in Table S1.

Although it was reassuring to see that text mining identified a number of established autophagy inhibitors, including bafilomycin A_1_, chloroquine, and hydroxychloroquine, a number of the small molecules in Table S1H are worthy of fresh attention. First, the “amino acids” entity was the most highly referenced negative modulator of autophagy after 3-methyladenine. Interestingly, arginine, asparagine, leucine, and phenylalanine were the only individual amino acids to make the list. Glutamine, as discussed later, is reported to be a dual modulator of autophagy. Another noteworthy negative modulator of autophagy is oxygen; indeed hypoxia in the tumor microenvironment may drive autophagy and promote tumorigenesis.^37^^-^^40^

Table S1I lists 268 small molecules reported to modulate autophagy positively. In general, most chemotherapeutic agents induce autophagy; tamoxifen, imatinib, and bortezomib, for example, were highly cited positive modulators. It was reassuring that the MTOR inhibitor rapamycin was identified as the top-ranked positive modulator of autophagy and that its clinical analogs everolimus and temsirolimus were also identified. The second highest-ranking positive modulator of autophagy was the entity “reactive oxygen species” (ROS). Damaged mitochondria are primary sources of ROS and, accordingly, are thought to induce a form of autophagy known as mitophagy to clear damaged mitochondria.^4^ Peroxide and nitric oxide, which interact with ROS to form reactive nitrogen species, are also highly referenced as positive modulators of autophagy. Further supporting the importance of ROS as a positive modulator, a number of antioxidants have been reported to inhibit autophagy, including acetylcysteine, ascorbic acid, butylhydroxyanisole, glutathione, lipoic acid, tiron (a cell-permeable superoxide scavenger), and vitamin E (Table S1H). Two final entities worth noting are the sphingolipids and ceramides, both of which are highly referenced as positive modulators of autophagy (Table S1I).

Table S1J lists 22 small molecules that both inhibit and stimulate autophagy according to text-mining results. If we exclude chemicals for which one of the relationships is supported by only 1 reference, 9 compounds were still identified as dual modulators of autophagy. How can a molecule both negatively and positively modulate autophagy? The following examples provide potential mechanisms. First, AICAR was originally described as an AMPK-dependent inhibitor of autophagy,^41^ but more recent work identifies AMPK-independent inhibition of autophagy by AICAR, possibly through inhibition of the class III PtdIns3K (whose catalytic subunit is PIK3C3/VPS34) to BECN1.^42^^,^^43^ Hence, cellular and genetic contexts appear to determine the direction of autophagy modulation by AICAR. Second, it is not surprising to find ATP as a positive modulator, since autophagy is an active process, but the conclusion that ATP negatively modulates the process lacks support. For example, one text reference included the phrase “autophagy is activated by a decrease in ATP,” which was inaccurately equated with negative modulation of autophagy by ATP. Since the primary mechanism by which ATP modulates autophagy involves activation of AMPK in response to a decrease of the ATP/AMP ratio,^44^ the authors could have prevented the text-mining error by writing “an increase in AMP” instead of “a decrease in ATP.” That said, ATP should theoretically feed back negatively on autophagy, since a primary function of autophagy is to generate energy to survive stress, and that function must be turned off when sufficient energy and nutrients have been generated. Third, glucose withdrawal has been extensively described to induce autophagy,^45^^,^^46^ but high glucose/hyperglycemia can also induce autophagy through MTOR^47^^-^^51^ and potentially through generation of ROS.^52^^-^^54^ A fourth entity, glutamine, is in the spotlight because of the many pathways in which it functions. Recently, the pathways that anabolize and catabolize glutamine (mediated by glutamine synthetase (GLUL) and glutaminase (GLS), respectively) have been found to modulate autophagy upstream of RRAG GTPases,^55^^,^^56^ implicating glutamine as a critical node in the modulation of autophagy (Fig. 3). Specifically, glutamine can positively modulate autophagy through glutaminolysis via the production of ammonia,^57^ a positive modulator of autophagy (Table S1I). Like other amino acids, however, glutamine negatively modulates autophagy through RRAG GTPases,^35^^,^^55^ MTOR signaling,^56^^,^^58^ and EIF2A-ATF4 signaling.^59^^-^^61^ Finally, metformin is another interesting small molecule reported to modulate autophagy both positively and negatively. Both inhibition and stimulation of autophagy by metformin appear to be AMPK-dependent,^62^^,^^63^ but the exact mechanisms are still under investigation. Overall, these and a number of additional chemicals listed in Table S1J appear to be dual modulators of autophagy, but in most cases additional studies would be required to define the molecular determinants and the contexts of such diverse behavior.

### Novel autophagy-based therapeutic strategies

After compiling an inventory of autophagy modulators, we wanted to leverage the integrated results to propose novel autophagy-targeted strategies for treating cancer. Inhibition of autophagy, in particular, has been reported to augment the efficacy of a number of therapeutic agents in preclinical studies.^64^ Therefore, there is a clear rationale for combining inhibitors of autophagy with other agents in clinical trials. A number of such trials are already underway,^65^ and the previous sections discussed a few new strategies worth testing. But based on new information from studies of the cell death pathway known as necroptosis, one trial is worth particular attention: evaluation of hydroxychloroquine (HCQ) (Table 2; Table S1H) in combination with the rapamycin (Table 2; Table S1I) analog temsirolimus. As single agents, allosteric TORC1 (Table 1; Table S1A) inhibitors like temsirolimus have shown limited activity in clinical trials,^66^ perhaps because autophagy is induced as a prosurvival mechanism. Another possible explanation for the limited clinical activity of rapalogs, however, is the finding that the primary executors of necroptosis, RIPK1 and RIPK3, are localized to mitochondria.^67^^,^^68^ That observation prompts the hypothesis that induction of mitophagy (i.e., autophagic degradation of mitochondria), which occurs in response to rapamycin treatment,^69^^,^^70^ degrades RIPK1 and RIPK3, thereby reducing the ability of cells to die through necroptosis. If that is true, combination of a TORC1 inhibitor (i.e., autophagy stimulator) like temsirolimus with an autophagy inhibitor like HCQ could sensitize cancer cells to necroptotic cell death. That has, indeed, been reported.^71^ On that basis, we propose the general hypothesis that combination therapies consisting of at least one mitophagy inhibitor and one non-mitochondrial autophagy stimulator might be useful in the treatment of cancer. The results of our analysis suggest additional autophagy inhibitors and stimulators worth evaluating in that regard (i.e., the positive and negative modulators listed in Table 2).

Another cancer treatment strategy that was prioritized by our integrated analysis of autophagy-modulating proteins and small molecules is based on targeting RELA (Table 1; Table S1G) for inhibition of autophagy. Text mining identified a report in which RELA activity was inhibited ~50% by treatment with the antioxidant acetylcysteine (Table S1H),^16^ and sesquiterpenes were also found to inhibit RELA,^17^ as mentioned previously. Hence, a nontargeted strategy using acetylcysteine or a sesquiterpene (e.g., helenalin) in combination with rapamycin may be worth evaluation as a proof-of-concept. A possible drawback of acetylcysteine therapy, however, could be toxicity associated with its ability to break disulfide bonds and disrupt redox homeostasis. The combination of rapamycin with acetylcysteine, however, might circumvent that possibility due to the antioxidant activity of acetylcysteine. A second therapeutic approach based on autophagy inhibition and the results of our analysis entails the use of oxygen. Hypoxia in tumor stroma has been observed to promote tumorigenesis and autophagy,^72^ prompting the hypothesis that pharmacological delivery of oxygen (e.g., using red blood cell as carriers^73^) could be useful in treatment of some cancers.

Our analysis also enabled us to prioritize 2 top candidate drug targets for stimulating autophagy—IGF1 (Table 1; Table S1F) and key amino acids (Table 2; Table S1H). As discussed previously, the MEK inhibitor UO126 is a candidate inhibitor of IGF1 that could be tested in combination with an autophagy inhibitor such as HCQ as a proof-of-concept prior to the development of molecules specifically targeted to IGF1. As for inhibition of amino acids, since our analysis identified asparagine and glutamine as important negative modulators of autophagy, and since ammonia is known to stimulate autophagy potently,^48^ evaluation of L-asparaginase as an autophagy stimulator is particularly interesting. L-asparaginase enzymatically releases ammonia from asparagine and glutamine in the process of catabolizing the 2 amino acids into aspartic acid and glutamic acid, respectively. Because L-asparaginase has been confirmed to induce autophagy,^74^ testing it in combination with HCQ would be a worthwhile proof-of-concept experiment. L-asparaginase has been used clinically since the 1970s to treat acute lymphoblastic leukemias.^75^^,^^76^

In special cases, inhibiting autophagy with single agents may be therapeutically effective. Aggressive tumors (e.g., those with constitutive RAS activation) have adapted to survive with high rates of autophagy and have been proposed to be “addicted” to autophagy.^77^ Therefore, inhibiting autophagy may decrease the tumorigenicity of RAS-expressing cancer cell line models.^77^^,^^78^ In support of that hypothesis, inhibition of autophagy by *atg7* knockout in *BRAF^V600E^-*driven or *KRAS^G12D^-*driven lung cancers altered tumor fate by diverting aggressive cancers to more benign disease.^79^^,^^80^ That observation suggests the possibility that increased RAS activation and inhibition of autophagy could be “synthetically lethal” in cancer patients or at least could significantly decrease tumor burden.

## Conclusion

Adequate autophagy-based therapeutic interventions for the treatment of cancer are currently lacking. Therefore, strategies that identify and analyze new modulators of autophagy may be useful. Here, we have integrated analyses of published siRNA screen data and pathway-based text-mining to construct an extensive inventory of genes, proteins, complexes, and small molecules that appear to modulate autophagy (Table S1). The inventory and analysis offer novel features: i) analysis and annotation of the direction (positive or negative) of autophagy modulation; ii) a semiquantitative (in the case of text-mined results) or quantitative (in the case of siRNA screen results) index for estimating the strength of evidence behind each entity reported as a modulator of autophagy; iii) a model of the autophagy pathway (Fig. 3) that incorporates new information from our analysis; iv) an indication of the possible utility, at least in concept, of combining an inhibitor of autophagy (e.g., an inhibitor of RELA) with a stimulator of autophagy (e.g., rapamycin or L-asparaginase), particularly if the inhibitor affects mitophagy and the stimulator affects a non-mitochondrial form of autophagy; v) Venn diagrams and associated quantitative analyses that indicate the sometimes surprising relationships (or lack thereof) among the different types of evidence in this complex, often confusing field. Indeed, since autophagy is being associated with ever-increasing numbers of other cellular processes, a future challenge will be to determine the specificity of all modulators (genes and compounds) that regulate autophagy, preferably in isogenic autophagy-wild-type and autophagy–deficient cells.

## Materials and Methods

### siRNA Screens

We compiled data from 4 published, macroautophagy-specific siRNA screens in human cell lines: siRNA screen 1 (753 siRNA pools targeting 705 genes)^8^ yielded 7 validated hits; siRNA screen 2 (21,121 siRNA pools targeting 16,492 genes)^9^ yielded 148 validated hits; siRNA screen 3 (726 individual kinase-targeted siRNAs)^10^ yielded 21 validated hits; and siRNA screen 4 (21,121 siRNA pools targeting 16,492 genes)^11^ yielded 169 validated hits. The data and explanatory details are provided in Table S2A. The Venn diagrams in Figure 1A (drawn to scale) show the sizes of the siRNA libraries and the numbers of hits in each screen. To achieve consistency throughout the analysis, it was necessary to pre-process the published data. For each screen, we i) translated redundant or outdated gene names or symbols into HUGO gene symbols, ii) excluded data in the rare cases in which we could not resolve naming ambiguities, and iii) assigned rank values to the genes based on number of references to provide a rough quantitative basis for comparing strength of evidence.

### Pathways and text mining

To complement the siRNA screen data, we used pathway analysis based on text-mining of the literature to identify autophagy modulators. The operational definition of “modulator” used here is rather broad: “an entity (gene, protein, protein complex or small molecule) that has been reported empirically to activate or inhibit autophagy.” Accordingly, knowledge of the mechanism was not required to list a particular entity as an autophagy modulator. That definition of “modulator” applies well to “hits” in siRNA screening, for which there is assumed to be an element of causality. The definition also applies to the major pathway analysis software packages, of which we compared 3: Ingenuity Pathway Analysis (IPA; Ingenuity Systems); MetaCore (Thomson Reuters GeneGo); and Pathway Studio (Elsevier/Ariadne Genomics). With IPA, we used the advanced search tool to search for the term “autophagy” as a function. That IPA search yielded 218 genes and 123 small molecules (Table S3). A similar approach using MetaCore yielded 38 genes. Both IPA and MetaCore use manually curated databases, so those hits can be considered in a sense to be validated. Pathway Studio, by contrast, uses automated text mining, which is more susceptible to false positives. Therefore, after using Pathway Studio to mine 10,087 PubMed abstracts and 228 full-text articles from a PubMed search for “autophagy,” we validated the Pathway Studio hits by manually curating (i.e., reading and analyzing) the text from which autophagy modulators were identified. The end result obtained with Pathway Studio was 421 genes and 385 small molecules, which are listed in Table S1. A more thorough description of the Pathway Studio method is provided in Supplementary Methods.

Uncurated pathway analysis based on text mining has its limitations. One is that the molecular definition of autophagy has changed over time and varies even among recent publications. Also, text-mining algorithms may incorrectly assign the direction (negative, unknown, or positive) of autophagy modulation, depending on whether the assay defined autophagy appropriately. For example, accumulation of MAP1LC3A (also known as LC3A) in autophagosomes has been invoked as an index of autophagy, but MAP1LC3A accumulation can reflect processes other than functional autophagic flux. It can also result from inhibition of autophagosome-lysosome fusion or autolysosome degradation, reflecting an abortive or defective autophagic process.^81^ To ensure the greatest possible accuracy of the directions assigned and the number of references that support each relationship, we manually curated all of the text-mined relationships by reading ~6,000 extracted text entries describing ~1,000 protein-autophagy and chemical-autophagy relationships. When a text-mining discrepancy was found, we assigned the direction of modulation, when possible, based on consensus in the autophagy field, which usually reflected the direction of modulation that occurs under nutrient-replete conditions. For example, MTOR is reported to modulate autophagy positively and separately reported to modulate it negatively (see Table S2A and Table S2B for references and exact sentences). We list MTOR as a negative modulator because it negatively modulates autophagy in the presence of sufficient nutrients. Table S2A and Table S2B contain a complete list of such ambiguities and their resolutions. As we did with siRNA data, the number of literature references that support each text-mined relationship was converted to a rank value to serve as a crude quantitative index for comparison across entities and their associations. We provide to-scale Venn diagrams and, in some instances, statistical analyses to aid the reader in assessing the robustness of evidence. See the legend of Figure 1 and Table S4 for explanations of the Venn diagram methodology.

### Data quality assessment

Although most of the human genome was covered by the combination of siRNA libraries (Fig. 1A), we discovered a number of limitations: i) only 2 of the screens (numbers 2 and 4) were “genome-wide” (16,492 genes);^9^^,^^11^ ii) as illustrated in Figure 1B, the siRNA screen hits exhibited little intersection with text mining hits; iii) despite the high degree of overlap among siRNA libraries, the hits in the different screens exhibited almost no intersection (Fig. 1A). The only overlap (out of a total of 342 hits) consisted of 3 genes in common between screens 2 and 4. Considering that the overlap expected by chance for the 2 independent screens is 1.52 genes, the enrichment over chance is modest (a factor of 1.98); iv) 3 of the 4 primary siRNA screens (i.e., initial screens as opposed to secondary, validation screens) measured autophagy using upstream markers such as MAP1LC3A-II accumulation or localization instead of downstream markers of autophagic flux (i.e., productive autophagy). The most recent, siRNA screen 4,^11^ was an exception; it employed a primary screen designed to distinguish hits that induce MAP1LC3A-II accumulation as a result of abortive autophagy from those that induce productive autophagy; v) measures of screen robustness (i.e., Z′-factors) were not reported for any of the screens, and the data necessary to calculate Z′-factors were not provided. Based on a cursory assessment, however, it is not clear that any of the 4 has a sufficiently large dynamic range and sufficiently low variance to yield robust Z′-factors.^82^ Together, those issues represent 2 types of limitations—ones that are general for siRNA screening, and others that are specific to interrogation of the autophagy pathway.

Pathway analysis also has limitations: i) although the intersections of hits among the 3 software packages IPA, MetaCore, and Pathway Studio were greater than the intersections among siRNA screens (Fig. 1B), a significant number of hits were unique to each software package; ii) as indicated above, the pathway analysis hits showed little intersection with siRNA screening hits. Because Pathway Studio yielded larger intersection with siRNA screening than did IPA or MetaCore (Fig. 1B), we chose Pathway Studio (with our manual curation) as the prime pathway analysis tool; iii) 19% (67/358) of the relationships identified by Pathway Studio were false positives with respect to our manually curated list (Table S2A). The manual curation also rescued 132 “unknown” relationships that would otherwise have been discarded, thereby increasing the number of apparently validated hits from 285 to 417.

Overall, neither siRNA screening nor pathway analysis appeared fully adequate to interrogate the universe of autophagy modulators. Therefore, we chose to focus on the union of validated siRNA and text-mined data sets (i.e., sets 1, 2, 3, 4, and 8 in Fig. 1B). That strategy yielded a more comprehensive census than any individual approach alone, but we provide sufficient data annotation in the **Supplementary Tables** so that the reader can choose instead to focus on candidates identified by any single siRNA or text-mining data set or any desired combination of intersections and/or unions of the sets. Since we cannot be fully certain whether the limitations reported here are specific to the autophagy pathway or whether they are technical limitations of the various approaches, future analyses of additional pathways will continue to shed light on the informatics issues. What we do know, however, is that the inconsistencies in designation of genes as positive or negative in their regulatory influence reflect uncertainties and context-dependent relationships in the field.

## Supplementary Material

Additional material

Additional material

Additional material

Additional material

Additional material

Additional material

## Abbreviations:

AICAR: 5-aminioimidazole-4-carboxamide ribonucleotide
AMP: adenosine monophosphate
ATP: adenosine triphosphate
GDP: guanosine diphosphate
GTP: guanosine triphosphate
GTPase: guanosine triphosphatase
HCQ: hydroxychloroquine
IPA: Ingenuity Pathway Analysis
PtdIns3P: phosphatidylinositol 3-phosphate
TORC1: target of rapamycin complex 1
ROS: reactive oxygen species
siRNA: short interfering ribonucleic acid
