# Supplementary material for: A curated census of autophagy-modulating proteins and small molecules: Candidate targets for cancer therapy
Source: Autophagy. 2014 May 12;10(7):1316–26. doi: 10.4161/auto.28773 (PMC4203555; doi:10.4161/auto.28773)
Supplement: Additional material [file auto-10-1316-s01.pdf]

Landes Bioscience www.landesbioscience.com

Supplemental Material to:

Philip L Lorenzi, Sofie Claerhout, Gordon B Mills, and John N Weinstein

A curated census of autophagy-modulating proteins and small molecules: Candidate targets for cancer therapy

Autophagy 2014; 10(7)
http://dx.doi.org/10.4161/auto.28773

www.landesbioscience.com/journals/autophagy/article/28773

Supplementary Methods. Details of methods for text mining-based pathway analysis

To identify autophagy-modulating genes, we used Pathway Studio 9.0 (Ariadne Genomics / Elsevier) to mine 10,087 PubMed abstracts and 228 full-text articles obtained by a PubMed search for "autophagy" (search performed on August 1, 2012). By PubMed's default settings, that search retrieved articles containing the term "autophagy" in All PubMed Fields, of which the most relevant are title, abstract, journal, and Medical Subject Heading (MeSH) terms (keywords assigned to each article by a subject analyst at NCBI). The abstracts and articles were processed using Pathway Studio's MedScan Reader tool and the ResNet Mammalian and ChemEffect cartridges. Each cartridge uses a slightly different text-mining algorithm; the ResNet Mammalian cartridge is optimized for extraction of information about mammalian genes, proteins, complexes, disease states, and small molecules, whereas the ChemEffect cartridge is optimized for extraction of information about small molecules. Extracted contents are stored in a local database in the form of entities and relationships. An entity can be a cell process, complex, disease, functional class, protein, small molecule, or treatment. A relationship is a link between two entities and is defined as one of the following types: regulation, chemical reaction, direct regulation, molecular transport, molecular synthesis, expression, binding, protein modification, promoter binding, or miRNA effect. Relationships are generally assigned a direction: positive, negative, or unknown (in the case of an ambiguous extraction).

After updating the ResNet 9.0 Mammalian database of entities and relationships by digesting abstracts and full-text articles, we added an "autophagy entity" (a "cell process") to a blank pathway. With the autophagy entity highlighted, we selected Add→Advanced→Neighbors of expansion = 1→upstream only. In the next "Set Filter Parameters" window, the following entities were included (boxes checked): "protein" and "complex." In the next "Set Filter Parameters" window, the following relationships were included (boxes checked): "regulation" and "direct regulation." After the database search was complete, all resulting entities and relationships were imported into Pathway Studio and saved as a protein-centric autophagy pathway. The process was repeated using the "small molecule" entity filter instead of "protein" and "complex," and the resulting small molecule-centric pathway was saved separately.

The initial searches yielded 547 proteins and 396 chemicals that modulate autophagy (**Tables S2A and S2B**, respectively). Those protein and chemical pathway relationships were manually curated by reading the sentence(s) from which each relationship was extracted (using the Relation Table view in Pathway Studio) and performing the following tasks: 1) attempting to assign a direction (positive or negative modulation) to all "unknown" relationships (i.e., those for which the sentence was too ambiguous for Pathway Studio to draw a clear conclusion); 2) deleting ambiguous sentences to which a direction could not be assigned; and 3) ensuring that the number of references supporting each relationship is accurate. Although such quality control dramatically improves the accuracy of the text-mined data, Step #2 can introduce false negatives. For example, Pathway Studio identified an "unknown" relation between the gene MTMR6 and autophagy, and because the extracted sentence was ambiguous, MTMR6 was filtered out of our analysis. Further investigation, however, indicated better sentences in the associated full text article that would have resulted in MTMR6 being included as a true negative modulator of autophagy. Reading the full text in that manner for each ambiguous entity would have significantly delayed the completion of this analysis. Hence, it was not possible to identify such entities systematically.

Tables S2A and S2B include complete lists of identified text-mining errors and the corresponding resolution. When relevant, discrepancies were resolved by favoring the relationship attributed to normal, nutrient-replete conditions (as opposed to stress or starvation conditions), as explained by way of the MTOR example in the full text.
